# Effects of Therapies Involving Plyometric-Jump Training on Physical Fitness of Youth with Cerebral Palsy: A Systematic Review with Meta-Analysis

**DOI:** 10.3390/sports12060152

**Published:** 2024-05-29

**Authors:** Exal Garcia-Carrillo, Rodrigo Ramirez-Campillo, Mikel Izquierdo, Ragab K. Elnaggar, José Afonso, Luis Peñailillo, Rodrigo Araneda, Daniela Ebner-Karestinos, Urs Granacher

**Affiliations:** 1 Exercise and Rehabilitation Sciences Institute, Faculty of Rehabilitation Sciences, Universidad Andres Bello, Santiago 7591538, Chile; exal.garcia@gmail.com (E.G.-C.); rodrigo.ramirez@unab.cl (R.R.-C.); luis.penailillo@unab.cl (L.P.); rodrigo.araneda@unab.cl (R.A.); daniela.ebner@unab.cl (D.E.-K.); 2 Navarrabiomed, Hospital Universitario de Navarra (HUN), Navarra Institute for Health Research (IdiSNA), Universidad Pública de Navarra (UPNA), 31008 Pamplona, Spain; mikel.izquierdo@gmail.com; 3 Department of Physical Therapy and Health Rehabilitation, Prince Sattam Bin Abdulaziz University, Al-Kharj 11942, Saudi Arabia; rke_pt2001@yahoo.com; 4 Department of Physical Therapy for Pediatrics, Faculty of Physical Therapy, Cairo University, Giza 12613, Egypt; 5 Centre of Research, Education, Innovation, and Intervention in Sport (CIFI2D), Faculty of Sport, University of Porto, 4200450 Porto, Portugal; jneves@fade.up.pt; 6 Department of Sport and Sport Science, Exercise and Human Movement Science, University of Freiburg, 79102 Freiburg, Germany

**Keywords:** plyometric exercise, human physical conditioning, movement, muscle strength, resistance training, exercise therapy, rehabilitation, motor activity, physical therapy modalities, youth sports, children

## Abstract

The aim of this systematic review was to assess the effects of plyometric-jump training (PJT) on the physical fitness of youth with cerebral palsy (CP) compared with controls (i.e., standard therapy). The PRISMA 2020 guidelines were followed. Eligibility was assessed using the PICOS approach. Literature searches were conducted using the PubMed, Web of Science, and SCOPUS databases. Methodological study quality was assessed using the PEDro scale. Data were meta-analyzed by applying a random-effects model to calculate Hedges’ g effect sizes (ES), along with 95% confidence intervals (95% CI). The impact of heterogeneity was assessed (*I*^2^ statistic), and the certainty of evidence was determined using the GRADE approach. Eight randomized-controlled studies with low-to-moderate methodological quality were included, involving male (n = 225) and female (n = 138) youth aged 9.5 to 14.6 years. PJT interventions lasted between 8 and 12 weeks with 2–4 weekly sessions. Compared with controls, PJT improved the muscle strength (ES = 0.66 [moderate], 95% CI = 0.36–0.96, *p* < 0.001, *I*^2^ = 5.4%), static (ES = 0.69 [moderate], 95% CI= 0.33–1.04, *p* < 0.001, *I*^2^ = 0.0%) and dynamic balance (ES = 0.85 [moderate], 95% CI = 0.12–1.58, *p* = 0.023, *I*^2^ = 81.6%) of youth with CP. Therefore, PJT improves muscle strength and static and dynamic balance in youth with CP compared with controls. However, more high-quality randomized-controlled trials with larger sample sizes are needed to provide a more definitive recommendation regarding the use and safety of PJT to improve measures of physical fitness.

## 1. Introduction

Cerebral palsy (CP) is a physically disabling condition, with an overall prevalence of 2.11 per 1000 live births [1]. CP occurs due to non-progressive disturbances in the developing fetal or infant brain until the age of two years [2,3]. CP often involves sensory, perceptual, cognitive, communication, and behavioral disturbances [4], which have a negative impact on daily life activities [5]. Moreover, CP encompasses a range of developmental motor disorders that predominantly affect posture and balance, as well as locomotion [6], leading to increased energy costs for walking [7] and reduced physical activity [8]. More than 40% of youth with CP face limitations in motor activities such as crawling, walking, running, or even playing [8]. Alongside reduced physical activity levels [8], CP has also been associated with impaired physical fitness, including cardiorespiratory fitness [9] and deficits in muscle power (e.g., rate of force development) [10,11], local muscle endurance [12], maximal muscle strength [12,13], and reduced linear sprint and change-of-direction speed [14]. Youth with CP also exhibit impaired body composition (e.g., reduced muscle mass) and muscle-tendon architecture [15,16,17], including longer and fewer sarcomeres in muscle contractures [18,19]. 

Neuro-rehabilitation regimes such as massage, conductive education, horseback riding, or neurodevelopmental treatment [20] are commonly used to treat the physical fitness of youth with CP [21], although some of these present low-to-poor effectiveness [22]. Recently, there has been growing research interest in the effects of physical exercise on CP-related consequences and various components of physical fitness [23,24,25] showing mainly improvements in muscle strength and endurance [22]. Resistance training appears to be an effective strategy to counteract the muscular weakness and balance/gait disorders of youth with CP [26,27,28,29,30,31,32,33]. However, interventions that solely focus on the promotion of maximal strength may have limited benefits for balance, mobility, gait, and activities of daily living [25,34,35,36]. Previously, researchers have reported that lower limb muscle power is strongly associated with mobility measures (e.g., gait, transfers, sports-related activities) of youth with CP at levels I and II of the Gross Motor Function Classification System (GMFCS) [10,37,38]. Accordingly, resistance training interventions targeting muscle power might be particularly important to improve balance, mobility, gait, and daily life activities that rely more on the rate of force (or torque) development than maximal strength [37,39].

Only a few systematic reviews have examined the effects of muscle power-related interventions on the physical fitness measures of youth with CP [40,41]. Findings from these reviews are heterogeneous, which makes it difficult to obtain a coherent understanding of the impact of power training on the physical fitness of youth with CP [34]. Furthermore, none of the reviews [40,41] utilized a meta-analytical approach, which hinders the availability of higher-level evidence for making clinical decisions [42]. Additionally, the existing review articles [40,41] did not include interventions involving plyometric-jump training (PJT), which may be a particularly valuable exercise method to enhance lower limb muscle power [43,44,45] and mobility, because walking and PJT exercises are characterized by coupled eccentric-concentric muscle activations. More specifically, PJT consists of multi-joint jump exercises that engage muscle activities utilizing the stretch-shortening cycle (SSC). PJT exercises using the SSC involve eccentric-concentric muscle action coupling with short (e.g., <250 ms) or long (e.g., ≥250 ms) foot-ground contact times [44,45,46]. PJT induces neuromuscular adaptations [43] that have functional relevance for youth with CP [47]. Previously, researchers were able to show that PJT-related training effects translate to improved everyday (walking) and sports-related activities (e.g., sprinting) [25,35,36,48]. Another advantage of PJT is its compatibility with other exercise protocols [43]. Indeed, PJT can be well-combined with other exercise types when included in multicomponent intervention programs, which is common in CP treatment [21,23,24,25]. The benefit of multicomponent intervention programs, including PJT, compared with single-mode intervention is the potential of multimodal exercise regimes to improve different components of physical fitness, such as cardiorespiratory fitness, muscle strength, power, and local muscular endurance [45,49,50,51,52,53,54,55,56,57,58,59,60,61,62]. Although PJT exercises can be physically demanding (i.e., requiring coordination and balance; high impact forces during landing), the exercises can be easily modified to suit the needs of youth with CP according to their initial level of physical fitness. The challenges derived from PJT for CP patients are significant but still manageable. CP often results in muscle spasticity, poor motor control, and balance issues [6], making high-impact and coordination exercises like PJT difficult. However, with proper modifications, such as adjusting the intensity, duration, and complexity of the exercises, PJT can be safely integrated into rehabilitation programs [63]. These modifications ensure that the exercises remain within the individual’s capabilities while still providing the benefits of improved muscle power, coordination, and overall physical fitness [55]. However, the effectiveness of PJT may vary depending on the subtype of CP, making it particularly suitable for individuals with unilateral or bilateral spastic CP at GMFCS levels I and II, where patients can walk without restrictions or with some limitations [64]. These subtypes are likely to benefit most from PJT due to their higher functional capacity and ability to perform jump-related exercises.

Over the past years, there has been a significant increase in the number of publications related to CP in the sports science literature. A basic search in the electronic database Web of Science using the syntax (WC = (sport sciences)) AND (TS = (cerebral palsy)) yielded 2,011 results, with 1,001 published articles in the last decade, including studies on PJT [41,44,45,55,65,66,67,68,69,70,71,72,73,74,75,76,77,78,79,80,81,82,83,84,85,86]. To date, no systematic review with meta-analysis has been conducted that examined the effects of PJT interventions on measures of physical fitness of youth with CP. Methodological variations among PJT studies conducted in youth with CP, such as differences in the type of PJT programming (e.g., training frequency, intensity, duration, exercise type), the assessed outcome measures (e.g., maximal strength), and patient characteristics (e.g., diplegia, hemiplegia), may have contributed to significant heterogeneity among the published articles. To address these methodological limitations (e.g., reduced sample size) and evaluate the extent of study heterogeneity, a systematic review is warranted and holds the potential to yield valuable insights [42]. Consequently, the primary aim of this systematic review with meta-analysis was to examine the effects of PJT on components of physical fitness in youth with CP compared with controls (i.e., youth with CP receiving standard therapy or alternative therapy [e.g., balance training]).

## 2. Materials and Methods

### 2.1. Registration

The protocol of this systematic review with meta-analysis was published on the Open Science Framework platform (OSF; project: https://osf.io/q4wb6 (accessed on 27 April 2023); registration: https://osf.io/dn8mh (accessed on 28 April 2023)) and in PROSPERO (code: CRD42023422271).

### 2.2. Procedures

A systematic review with meta-analysis was conducted following the guidelines of the Preferred Reporting Items for Systematic Reviews and Meta-Analyses (PRISMA 2020) [87,88] and other pertinent recommendations in the field [44,45,89].

### 2.3. Inclusion and Exclusion Criteria

To ensure the inclusion of relevant studies, a PICOS (participants, intervention, controls [comparators], outcomes, and study design) approach was used to assess the eligibility of studies, as recommended by the PRISMA guidelines [88]. The specific inclusion and exclusion criteria are outlined in Table 1. This meta-analysis included original, peer-reviewed, full-text studies written in English, Spanish, or German.

### 2.4. Literature Search: Administration and Update

Computerized literature searches were conducted in the electronic databases PubMed, Web of Science, and SCOPUS. To develop the search strategy for PJT-related studies conducted in participants with “cerebral palsy,” the Boolean operators AND/OR were used in different combinations with keywords, such as “ballistic,” “complex,” “cycle,” “explosive,” “force,” “plyometric,” “shortening,” “stretch,” “training,” “velocity,” “jump,” “power.” The Supplementary Table S1 provides examples of search combinations and the search strategy code line for each database. 

The literature search was conducted with the aim of identifying all eligible studies from inception up to June 2023. The same author (R.R.-C.) conducted the initial search and removed duplicates, and two authors (R.R.-C. and E.G.-C.) independently screened the titles, abstracts, and full-text versions of the retrieved articles. Any potential discrepancy between the two authors regarding inclusion and exclusion criteria was resolved through consensus with a third author (R.K.E.). Reference lists of selected articles were analyzed to identify any additional relevant studies.

### 2.5. Data Collection Process

Means and standard deviations of dependent variables were extracted from the included studies at pre- and post-PJT time points using custom-made Microsoft Excel sheets (version 16.66.1, Microsoft Corporation, Redmond, WA, USA). Two authors (E.G.-C. and R.R.-C.) performed data extraction independently, and any discrepancies between them (e.g., mean value for a given outcome) were resolved through consensus with a third author (M.I.).

### 2.6. Data Items

The extraction of dependent variables from the included studies considered previous recommendations and definitions [94] regarding relevant outcomes for participants with CP [11,92]. Reliability measures, such as the intra-class correlation coefficient, were extracted when reported. The main outcomes were measures of physical fitness (e.g., muscle strength, balance), which are reliable [95] and essential to ensuring strong consistency between the analyzed studies within a meta-analysis [87]. 

Data extracted regarding the PJT programming parameters included (i) the box height used during PJT exercises; (ii) whether the PJT was combined with another lower-limbs training method; (iii) duration (number of weeks) of the PJT intervention; (iv) weekly PJT frequency; (v) intensity of the PJT exercises; (vi) number of total jumps completed during the PJT intervention; (vii) progressive overload applied during the PJT intervention; (viii) recovery time between sets, repetitions, and training sessions; (ix) replacement of a given part of the standard sport training schedule (if applicable) with PJT exercises; (x) type of PJT exercises; and (xi) type of surface used during PJT. We also extracted data regarding participants’ sex, age (years), body mass (kg), height (m), previous experience with PJT, type, and level of sport practiced (if applicable).

### 2.7. Methodological Quality Assessment

The methodological study quality was assessed using the PEDro scale, a reliable and previously validated tool [96,97]. This tool has frequently been used in systematic reviews in this field [45,89,98,99,100] and in the context of PJT used during rehabilitation [101]. The tool enables comparisons between studies and assists readers in judging the reliability and clinical relevance of the trial results. The methodological study quality was classified as high (≤3 points), moderate (4–5 points), and low (6–10 points). Two authors (R.K.E. and R.R.-C.) assessed each study independently, and discrepancies were resolved through consensus with a third author (E.G.-C.).

### 2.8. Summary Measures, Synthesis of Results, and Publication Bias

Meta-analyses can be conducted with as few as two studies [102]. Nonetheless, a meta-analysis was performed only when at least three studies were available [103,104], given that reduced sample sizes are common in the sport-science literature [105], including PJT studies [44,45,106,107]. Hedges’ g effect sizes (ES) were calculated for each outcome attribute in the PJT and control groups using pre-training and post-training means and standard deviations. Post-intervention standard deviation values were used to standardize the data. The DerSimonian and Laird inverse random-effects model was used to account for differences between studies that could affect the PJT effect [108,109]. The calculated ES values were presented with 95% confidence intervals (95% CIs) and interpreted using the following scale: <0.2 trivial, 0.2–0.6 small, >0.6–1.2 moderate, >1.2–2.0 large, >2.0–4.0 very large, >4.0 extremely large [110]. When a study included more than one intervention group and a single control group, the sample size in the control group was proportionally divided to facilitate comparisons across multiple groups [111]. The impact of study heterogeneity was assessed using the *I*^2^ statistic, with values of <25%, 25–75%, and >75% representing low, moderate, and high levels of study heterogeneity, respectively [112]. A sensitivity analysis (automated leave-one-out analysis) was conducted to assess the robustness of the summary estimates (e.g., *p*-value). The Comprehensive Meta-Analysis software (version 2, Biostat, Englewood, NJ, USA) was used for analyses, with *p* < 0.05 as the level of statistical significance.

### 2.9. Certainty of Evidence

Two authors, J.A. and R.R.-C., used the Grading of Recommendations Assessment, Development and Evaluation (GRADE) to determine the level of evidence certainty for each outcome [113,114,115]. Evidence started out as having high certainty and was downgraded based on the following criteria: (i) risk of bias in studies, with an average PEDro score of 4–5 points being downgraded by one level, and ≤3 points by two levels; (ii) indirectness, which was considered low risk due to the specificity of the populations, interventions, controls, and outcomes outlined in the eligibility criteria; (iii) the risk of publication bias, with a suspected publication bias resulting in a one-level downgrade; (iv) inconsistency, with a moderate impact of statistical heterogeneity (*I*^2^ ≥ 25%) resulting in a one-level downgrade and a high impact (*I*^2^ > 75%) resulting in a two-level downgrade; and (v) imprecision, with a one-level downgrade occurring if the comparison included less than 800 participants and/or if no clear direction of effects was noted [116]. In cases where both imprecision criteria were observed, the level of certainty was downgraded by two levels. For outcomes where the number of comparison trials was insufficient for a meta-analytical approach, the evidence was judged to be of very low certainty.

## 3. Results

### 3.1. Study Selection

**Figure 1:**
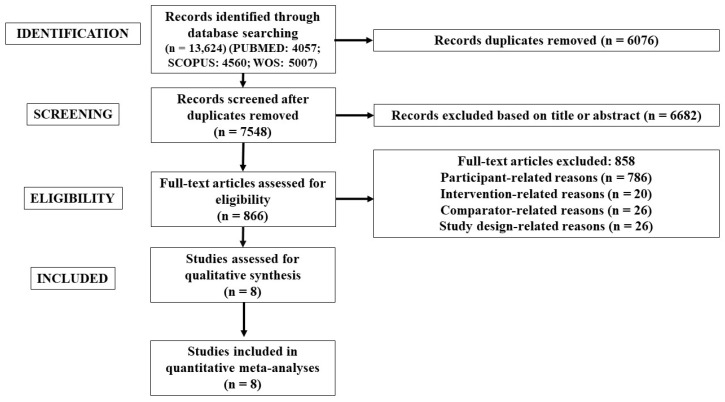
Flow chart illustrating the study selection process.

The search conducted in the databases yielded a total of 13,624 records. The study selection process is depicted in Figure 1 (adapted from the PRISMA guidelines), illustrating the flow of inclusion and exclusion of studies. Following the removal of duplicate records (n = 6076), a screening of titles and abstracts resulted in the exclusion of 6682 records. Subsequently, a thorough evaluation of 866 full texts was conducted. Ultimately, eight studies [55,67,70,71,72,117,118,119] were deemed eligible for inclusion in the systematic review with meta-analysis.

### 3.2. Methodological Quality of the Included Studies

According to the PEDro checklist results (Table 2), the median score was 8.0, indicating a low risk of bias. From the eight included studies, seven achieved scores ranging from seven to eight points, indicating a low risk of bias. One study received a score of four points, indicating a moderate risk of bias. It is worth noting that all eight studies followed supervised exercise interventions, with four studies indicating a 1:1 therapist-to-participant ratio, ensuring a controlled and supervised environment for the participants.

### 3.3. Study Characteristics

Table 3 provides an overview of participants’ characteristics and the intervention programs used in the included studies. The eight included randomized-controlled studies that comprised ten intervention groups and eight control groups. Among the control groups, seven received standard therapy [67,70,71,72,117,118,119], and one control group received alternative therapy with a focus on balance training [55]. The standard therapy usually included a mix of strength, balance, gait, mobility, and stretching exercises. Our quantitative analyses included a total of 363 participants, with 205 in the intervention group and 158 in the control group. The ages of participants ranged from 9.5 to 14.6 years. All included studies had mixed samples of both male and female participants, with 225 males and 138 females in total. The duration of the intervention programs ranged from 8 to 12 weeks, with most of the studies lasting 12 weeks (62.5%, n = 5). The weekly training frequency varied from two to four sessions (25–60 min), with most studies applying two weekly sessions (75%, n = 6).

### 3.4. Results from the Meta-Analyses

#### 3.4.1. Muscle Strength

**Figure 2:**
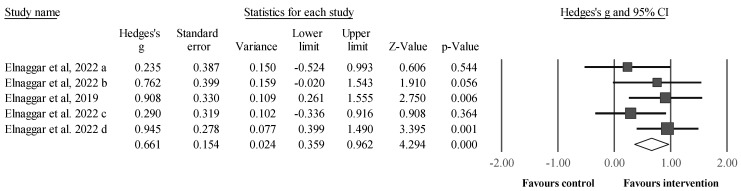
Forest plot illustrating plyometric jump training-related improvements in muscle strength compared with controls. Forest plot values are shown as effect sizes (ES [Hedges’ g]) with 95% confidence intervals (CI). Black squares: individual studies. The size represents the relative weight. White rhomboid: summary value. a, b, c, d: denotes different trials published in the same year, led by the same author; except “a” and “b”, denoting the inclusion of two experimental groups in one trial.

Four studies provided data for muscle strength (Figure 2), involving five intervention groups and four control groups. Maximal strength was measured isometrically (quadriceps, hamstrings, plantar dorsiflexors, and flexors) and dynamically (squat). Results showed a significant and moderate effect for the intervention compared with the control (i.e., three receiving standard therapy, one receiving alternative therapy [balance training]) groups: ES = 0.66, 95% CI = 0.36 to 0.96, *p* < 0.001, *I*^2^ = 5.4%, total participants n = 190. After the sensitivity analyses (automated leave-one-out analysis), the robustness of the summary estimates (e.g., *p*-value) was confirmed.

#### 3.4.2. Static Balance

**Figure 3:**
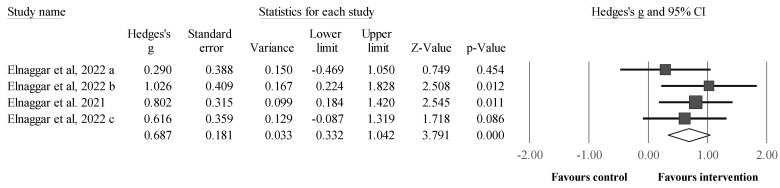
Forest plot illustrating plyometric jump training-related improvements in measures of static balance compared with controls. Forest plot values are shown as effect sizes (ES [Hedges’ g]) with 95% confidence intervals (CI). Black squares: individual studies. The size represents the relative weight. White rhomboid: summary value. a, b, c: denotes different trials published in the same year, led by the same author; except “a” and “b”, denoting the inclusion of two experimental groups in one trial.

Three studies provided data for static balance (Figure 3), involving four intervention and three control groups. Static balance was measured in the antero-posterior direction and the medio-lateral direction under stable and unstable surface conditions. Results showed a significant and moderate effect for the intervention compared with the control group (i.e., two receiving standard therapy, one receiving alternative therapy [balance training]) groups: ES = 0.69, 95% CI = 0.33 to 1.04, *p* < 0.001, *I*^2^ = 0.0%, total participants n = 130. No sensitivity analyses (automated leave-one-out analysis) were performed as per the reduced number of studies available to assess the robustness of the summary estimates (e.g., *p*-value).

#### 3.4.3. Dynamic Balance 

**Figure 4:**
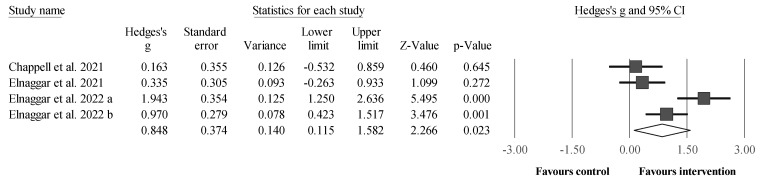
Forest plot illustrating plyometric jump training-related improvements in measures of dynamic balance compared with controls. Forest plot values are shown as effect sizes (ES [Hedges’ g]) with 95% confidence intervals (CI). Black squares: individual studies. The size represents the relative weight. White rhomboid: summary value. a, b: denotes different trials published in the same year, led by the same author.

Four studies provided data for measures of dynamic balance (Figure 4), involving four intervention groups and four control groups. Dynamic balance was measured using the end-point maximal excursion test, the maximal limit of stability test, the maximal antero-posterior sway tests, and the maximal antero-direction test. Results showed a significant and moderate effect of the intervention compared with the control groups (i.e., all four receiving standard therapy): ES = 0.85, 95% CI = 0.12 to 1.58, *p* = 0.023, *I*^2^ = 81.6%, total participants n = 175. After the sensitivity analyses (automated leave-one-out analysis), with the removal of one study [119], the results also showed a moderate effect favoring the intervention compared with the control groups, although without reaching the level of statistical significance: ES = 0.81, 95% CI = −0.27 to 1.89, *p* = 0.142, *I*^2^ = 87.5%.

### 3.5. Additional Analyses

#### 3.5.1. Certainty of Evidence

Results from the GRADE analyses are presented in Table 4. For muscle strength and static balance, the certainty of evidence is considered moderate, and for dynamic balance, it is rated as very low.

#### 3.5.2. Adverse Effects

One study reported that there were no adverse health events due to the intervention program [118]. Seven studies did not mention unwanted side effects due to the intervention, including injury, pain, delayed onset of muscle soreness, undue fatigue, or related adverse events. Drop-outs in the intervention groups were <14% [55,67,70,71,72,117,118,119], and none of the dropouts were associated with PJT-related adverse effects. When reported [55,118], compliance with the PJT interventions varied between 75–100% (mean values >90%) of the scheduled treatment sessions.

## 4. Discussion

The primary objective of this systematic review with meta-analysis was to examine the effects of PJT on measures of physical fitness in youth with CP compared with controls. Eight randomized-controlled trials with a low-to-moderate risk of bias were eligible for inclusion, involving males (n = 225) and females (n = 138) aged 9.5 to 14.6 years. The duration of the intervention programs ranged from 8 to 12 weeks, with two to four weekly sessions. The PJT was either added to the therapy received by the controls [70,71,72,117,118,119] or replaced the therapy received by the controls [55,67]. In seven out of the eight included studies, the control groups received standard therapy, involving a mix of strength, balance, gait, mobility, and stretching exercises; in one study, the control group received alternative therapy with an emphasis on balance exercises. Compared with controls, PJT interventions resulted in significant improvements in muscle strength (four studies; ES = 0.66 [moderate], 95% CI = 0.36–0.96, *p* < 0.001, *I*^2^ = 5.4%), static balance (three studies; ES = 0.69 [moderate], 95% CI = 0.33–1.04, *p* < 0.001, *I*^2^ = 0.0%), and dynamic balance (four studies; ES = 0.85 [moderate], 95% CI = 0.12–1.58, *p* = 0.023, *I*^2^ = 81.6%) among youth with CP. The certainty of the evidence ranged from moderate (muscle strength; static balance) to very low (dynamic balance). 

Compared with controls, 8 to 12 weeks of PJT resulted in significant muscle strength improvements among male and female youth with CP. These results confirm the positive effects of PJT on maximal strength reported in previous studies involving various participant groups [120,121]. The enhancement in maximal strength following PJT may be attributed to several underlying mechanisms, including greater motor unit recruitment [122], improved muscle activation strategies (e.g., improved intermuscular coordination) [43], improved single-fiber functioning (e.g., increased force) [123,124], increased muscle mass [123,124,125,126,127], and improved muscle-tendon architecture (e.g., increased muscle pennation angle) [43,125]. As youth with CP often exhibit reduced levels of muscular strength and power [10,11], local muscular endurance [12], maximal muscle strength [12,13], and the underlying physiological mechanisms such as reduced muscle mass and/or muscle-tendon architecture [15,16,17], an increased level of muscle strength is expected to be beneficial. However, based on the GRADE assessment (Table 4), the certainty of the evidence was moderate, and the available body of evidence is limited to eight studies. Future studies should aim to generate more robust evidence by including larger samples in randomized-controlled trials. Moreover, the PJT interventions in the included studies did not extend beyond 12 weeks, with measurements taken at 8 and 12 weeks. Therefore, further research is needed to investigate the long-term effects of PJT on muscle strength in youth with CP and to explore whether early improvements (<8 weeks) are possible.

Youth with CP commonly experience deficits in static and dynamic balance [7,128]. These motor deficiencies are usually treated through more traditional neuro-rehabilitation techniques [5,21] such as Bobath and Vojta methods [129], despite their proven lack of effectiveness [5,22]. More recent rehabilitation methods have received increased research attention due to their promising effects [23,24,25]. For example, resistance training has emerged as an effective strategy for treating muscle weakness and balance/gait disorders in youth with CP without exacerbating spasticity [26,27,28,29,30,31,32,33]. Indeed, the results obtained from our meta-analysis demonstrate that PJT as a single intervention or included in multicomponent interventions can lead to improved static and dynamic balance (ES = 0.69 and 0.85, respectively). However, the certainty of the evidence was very low regarding dynamic balance. These findings are consistent with previous studies that have shown the positive effects of PJT on balance in non-CP subjects [100]. It has been speculated that PJT may stimulate several physiological mechanisms, particularly during jump landings, which could be associated with the observed improvements in balance [100]. These mechanisms include anticipatory postural adjustments, proactive muscle activation (pre-innervation) prior to landing, enhanced sensitivity of afferent feedback loops, co-activation of lower limb muscles, proprioception, and neuromuscular control [100]. Muscle weakness and impaired balance control are frequently cited as contributing factors to walking difficulties in adults with CP [130]. Therefore, in addition to enhancing muscle strength, improved PJT-related balance may potentially assist youth with CP in walking. It is worth noting that over 40% of youth with CP aged three to 17 years face limitations in crawling, walking, running, or playing [8], indicating that a significant portion of the CP population may benefit from PJT interventions. Moreover, CP encompasses a group of developmental disorders that have a negative impact on posture and movement, including locomotion, leading to reduced levels of physical activity [8]. Therefore, PJT-related improvements in balance and muscle strength may translate to improved mobility [130] and increased physical activity, thereby reducing the risks associated with physical inactivity commonly observed in youth with CP [131]. 

### Limitations and Future Research Directions

Potential limitations and directions for future research should be discussed regarding the findings of this systematic review with meta-analysis. First, the sample sizes of the included studies were rather small. This highlights the need for larger-scale randomized controlled trials to provide more robust evidence regarding the effects of PJT on measures of physical fitness in youth with CP. Second, the limited number of high-quality studies (e.g., randomized controlled trials) may have reduced the overall robustness of the meta-analysis. Particularly, the meta-analysis regarding dynamic balance involved only four studies, and it showed a significant and moderate effect for the intervention groups compared with the control groups. However, a sensitivity analysis revealed that removing one study resulted in a loss of the significance level of the respective finding, although the effect size remained constant. Third, only three main outcome measures were included in the meta-analyses. As per the GRADE protocol, outcomes that were not analyzed attained a very low certainty of evidence. Therefore, at present, a robust recommendation regarding the use and safety of PJT to improve the various aspects of physical fitness in youth with CP is not feasible. Fourth, only three variables from a single domain of the International Classification of Functioning, Disability, and Health were analyzed, namely muscle strength and static and dynamic balance. This focused analysis may limit the comprehensive understanding of the impact of PJT on other aspects of physical fitness and functioning in youth with CP (e.g., pain perception, range of movement, mobility). Particularly, future studies may explore whether PJT has the potential to improve gait parameters (e.g., kinematics, kinetics, and electromyographic activity). Five, the ecological validity of PJT may be questioned for some youth with CP. For example, according to the GMFCS 5-level scale, individuals with CP can be categorized as level I (walk without restrictions), level II (walk with limitations), level III (walk using a manual gait aid), level IV (limited self-mobility; may use motorized mobility), and level V (transported in a wheelchair). PJT can most likely be performed on individuals with unilateral or bilateral spastic CP, but only at GMFCS levels I and II. 

In line with the previous arguments, there are different ways to define CP subtypes, including tonus-related (hypertonia [spastic], dystonia, hypotonia), topography-based (unilateral [hemiplegia, monoplegia], bilateral [diplegia, quadriplegia]), and gross motor function-based (GMFCS 5-level scale). Moreover, the classifications or subtypes of CP can be intertwined. According to our study protocol, additional analyses, such as moderator analyses and meta-regression analyses, were planned. However, due to the limited number of trials (≤4) per analyzed outcome measure, these additional analyses could not be conducted. Further, subgroup analyses regarding how PJT may operate differently depending on the subtype of CP were precluded due to the limited number of available studies. Future studies should address the feasibility, safety, and effectiveness of PJT in youth with a particular CP subtype while also considering their level of gross motor function. This would be particularly relevant if long-term studies are considered. Indeed, the studies included in this systematic review lasted no more than 12 weeks. Although the evidence observed in this review demonstrates a positive effect of PJT interventions in youth with CP, it is necessary to clarify whether this improvement persists in longer-term studies due to the reduced level of physical activity observed in children with CP [132]. Additionally, it is still uncertain whether the observed improvements can be transferred to other important aspects of the rehabilitation process, such as daily life activities or social participation, and how this transfer compares with therapy that has demonstrated efficacy in transferring motor improvements into daily life activities (e.g., goal-directed training), aspects that have been underconsidered in resistance training studies [32].

## 5. Conclusions

PJT, compared with standard therapy (e.g., stretching), has the potential to improve measures of physical fitness such as muscle strength as well as static and dynamic balance in youth with CP. However, due to the identified limitations, such as small sample sizes and a limited number of analyzed outcomes, a robust recommendation for the implementation of PJT to improve physical fitness in youth with CP cannot yet be made. Additionally, while PJT appears to be feasible, safe, and acceptable for youth with CP, formal assessment of these elements is lacking in published studies. Therefore, more high-quality randomized-controlled trials with larger sample sizes are needed to provide a more definitive recommendation regarding the use and safety of PJT to improve measures of physical fitness in youth with CP. 

## Figures and Tables

**Table 1:** Selection criteria used in the meta-analysis according to the PICOS approach.

Category	Inclusion Criteria	Exclusion Criteria
Population	Studies that included male and female participants with cerebral palsy aged ≤18 years, with no restrictions concerning the level of physical fitness or the sport practiced.	Studies that included participants aged >18 years or individuals with comorbidities to cerebral palsy [90,91] considered a contraindication [92] and/or precluding to engage fully (e.g., maximal or near-maximal effort) in a plyometric-jump training program (e.g., acute musculoskeletal injuries, recent surgery).
Intervention	Studies that included a plyometric-jump training program lasting ≥2 weeks with at least ≥6 total training sessions (i.e., minimal effective dose) [93], which included unilateral and/or bilateral jump exercises, loaded or unloaded, with repeated (cyclical) or non-repeated (non-cyclical) jumps, which commonly utilize long (countermovement jump) or short (drop jump) stretch shortening cycles.	Studies that included exercise interventions not involving plyometric-jump training (e.g., upper-body plyometrics only) or exercise interventions involving plyometric-jump training programs representing less than 50% of the total dedicated-intervention training load (i.e., lower-limbs number of exercises) when delivered in conjunction with other training interventions (e.g., high-load resistance training).
Control (comparator)	Studies that included active (e.g., standard therapy), specific-active (e.g., alternative therapy; regular sport practice), or passive control groups, involving participants with or without cerebral palsy.	Studies without a control group.
Outcome	Studies reporting at least one measure related to physical fitness (e.g., jump height-distance or related jump measure [e.g., force; power]; body composition; muscle strength; asymmetries; rate of force or torque development) ^a^ before and after the training intervention.	Studies without baseline and/or follow-up physical fitness data.
Study design	Experimental studies using (randomized) controlled designs.	Single-group interventions, no controls.

^a^: physical fitness as per the definition of Caspersen et al. [94].

**Table 2:** The Physiotherapy Evidence Database (PEDro) scale.

Criteria	Reviewed Studies							
	Chappell et al. [67]	Elnaggar et al. [55]	Elnaggar et al. [72]	Elnaggar et al. [71]	Elnaggar et al. [118]	Elnaggar et al. [117]	Elnaggar et al. [119]	Elnaggar et al. [70]
Eligibility criteria were specified.	YES	YES	YES	YES	YES	YES	YES	YES
Subjects were randomly allocated to groups.	YES	YES	YES	YES	YES	YES	YES	YES
Allocation was concealed.	NO	YES	YES	YES	YES	YES	YES	YES
The groups were similar at baseline regarding the most important prognostic indicators.	YES	YES	YES	YES	YES	YES	YES	YES
There was blinding of all subjects.	NO	NO	NO	NO	NO	NO	NO	NO
There was blinding of all therapists who administered the therapy.	NO	NO	NO	NO	NO	NO	NO	NO
There was blinding of all assessors who measured at least one key outcome.	NO	YES	YES	YES	YES	YES	YES	YES
Measures of at least one key outcome were obtained from more than 85% of the subjects initially allocated to groups.	NO	YES	YES	YES	YES	YES	YES	YES
All subjects for whom outcome measures were available received the treatment or control condition as allocated or, where this was not the case, data for at least one key outcome was analysed by “intention to treat”.	NO	YES	NO	YES	YES	YES	YES	NO
The results of between-group statistical comparisons are reported for at least one key outcome.	YES	YES	YES	YES	YES	YES	YES	YES
The study provides both point measures and measures of variability for at least one key outcome.	YES	YES	YES	YES	YES	YES	YES	YES
Total PEDro score	4/10	8/10	7/10	8/10	8/10	8/10	8/10	7/10

Note: The criteria “Eligibility criteria were specified”, related to external validity (i.e., “generalizability” or “applicability” of the trial), has not been used in calculation of the PEDro score.

**Table 3:** Descriptive characteristics of the youth cerebral palsy individuals participating in plyometric jump training interventions.

Study	Rand	Sex	Age (years)	Body Mass (kg)	Height (cm)	Fr	Weeks	CP Classification Declared	Int	Facilitator	Environment	PJT Exercises
Chappell et al. [67]	Yes	Mix	12.9	NR	NR	4	12	GMFCS levels I and II	NR	Physiotherapist	Group setting and home program	Jump, hop, run
Elnaggar et al. [72]	Yes	Mix	9.5	34.6	134	2	8	Unilateral	High	Pediatric physiotherapist	Structured clinical setting	Bound, Forward jump, Forward hop, Counter jump, Lateral leap, Stride jump, Squat jump, Tuck jump, Step jump, Step hop
Elnaggar et al. [71]	Yes	Mix	10.4	42.5	141	2	8	Unilateral	NR	Pediatric physiotherapist	Structured clinical setting	Bound, Forward jump, Forward hop, Counter jump, Lateral leap, Stride jump, Squat jump, Tuck jump, Step jump, Step hop
Elnaggar et al. [55]	Yes	Mix	10.0/10.0 ^a^	35.2/34.6	131/132	2	8	Spastic hemiplegic	High	Pediatric physiotherapist	Structured clinical setting	Bound, Forward jump, Forward hop, Counter jump, Lateral leap, Stride jump, Squat jump, Tuck jump, Step jump, Step hop
Elnaggar et al. [70]	Yes	Mix	10.6	43.3	142	2	12	Hemiplegic	High	Pediatric physiotherapist	Structured clinical setting	Bound, Forward-jump, Single-leg forward hop, Lateral leap, Side-to-side jump, Reciprocal stride-jump, Squat-jump, Tuck-jump, High-step hop, High-step jump
Elnaggar et al. [117]	Yes	Mix	13.0	47.5	149	2	12	Unilateral	High	Pediatric physiotherapist	Structured clinical setting	Bound, Forward-jump, Single-leg forward hop, Lateral leap, Side-to-side jump, Reciprocal stride-jump, Squat-jump, Tuck-jump, High-step hop, High-step jump
Elnaggar et al. [118]	Yes	Mix	14.6/14.5	50.3/51.8	152/154	2	12	Unilateral	High	Pediatric physiotherapist	Structured clinical setting	Lateral push-off, Jump split squat, SLVJ, Single-leg tuck jump, Double-leg hop, Side-to-side jump, Reciprocal stride-jump, Double-leg vertical jump, Double-leg tuck jump
Elnaggar et al. [119]	Yes	Mix	13.4	42.6	141	3	12	Hemiparetic	High	Physiotherapist	Structured aquatic setting	Ankle hop, Single-leg hop, Tuck jump, Countermovement jump, Lateral jump, Standing long jump, Drop jump, Box jump, One-leg jump vertical, One-leg jump lateral

Abbreviations ordered alphabetically: CP: cerebral palsy; Fit: fitness; Fr: frequency of weekly plyometric jump training sessions; GMFCS: gross motor function classification scale; Int: intensity (i.e., most studies reported a high jump training intensity, although without quantification); NR: no reported; Rand: randomized; PJT: plyometric jump training; SLVJ: single-leg vertical jump. ^a^: Values reported as XX.X/XX.X denotes those when two experimental groups were included.

**Table 4:** GRADE analyses.

Outcomes *	Number of Studies (PSS)	Risk of Bias in Studies	Risk of Publication Bias	Inconsistency	Imprecision	Certainty of Evidence
Muscle strength	4 (n = 190)	No downgrading	Not applicable	No downgrading (*I*^2^ = 5.4%)	Downgrade by one level: (i) <800 participants; (ii) moderate effect favoring PJT	⊕⊕⊕ Moderate
Static balance	3 (n = 130)	No downgrading	Not applicable	No downgrading (*I*^2^ = 0.0%)	Downgrade by one level: (i) <800 participants; (ii) moderate effect favoring PJT	⊕⊕⊕ Moderate
Dynamic balance	4 (n = 175)	No downgrading	Not applicable	Downgraded by two levels (*I*^2^ = 81.6%)	Downgrade by one level: (i) <800 participants; (ii) moderate effect favoring PJT	⊕ Very low

(i) Risk of bias in studies: downgraded by one level if the median PEDro scores were <6 or by two levels if ≤3; (ii) Indirectness: considered low due to eligibility criteria; (iii) Risk of publication bias: not assessed, as all comparisons had <10 studies available; (iv) Inconsistency: downgraded by one level when the impact of statistical heterogeneity (*I*^2^) was moderate (>25%) and by two levels when high (>75%); (v) Imprecision: downgraded by one level when <800 participants were available for a comparison or if there was no clear direction of the effects; accumulation of both resulted in downgrading by two levels. GRADE: Grading of Recommendations Assessment, Development and Evaluation; PJT: Plyometric jump training; PSS: pooled sample size; *: interventions vs. controls. ⊕: one, two, three, and four symbols denotes very low, low, moderate, and high certainty of evidence, respectively.

## Data Availability

All data generated or analyzed during this study will be included in the article as Table(s), Figure(s), and/or Electronic Supplementary Material(s). Any other data requirement can be directed to the corresponding author upon reasonable request due to privacy and ethical restrictions.

## Supplementary Materials

The following supporting information can be downloaded at: https://www.mdpi.com/article/10.3390/sports12060152/s1, Table S1: Search strategy (code line) for each database.
