# Supplementary material for: Effects of Therapies Involving Plyometric-Jump Training on Physical Fitness of Youth with Cerebral Palsy: A Systematic Review with Meta-Analysis
Source: Sports (Basel). 2024 May 29;12(6):152. doi: 10.3390/sports12060152 (PMC11207881; doi:10.3390/sports12060152)
Supplement: Supplementary file 1 [file sports-12-00152-s001.zip › sports-3001483-supplementary.pdf]

Electronic Supplementary Material Table S1

Article title: Effects of therapies involving plyometric-jump training on physical fitness of youth with cerebral palsy: A systematic re-view with meta-analysis

Author names: Exal Garcia-Carrillo, Rodrigo Ramirez-Campillo, Mikel Izquierdo, Ragab K. Elnaggar, José Afonso, Luis Peñailillo, Rodrigo Araneda, Daniela Ebner-Karestinos, Urs Granacher

Affiliation and e-mail of the corresponding author:
Prof. Urs Granacher, PhD
University of Freiburg, Department of Sport and Sport Science, Exercise and Human Movement Science, Sandfangweg 4, 79102 Freiburg i. Br., Germany.
Email: urs.granacher@sport.uni-freiburg.de; ORCID: 0000-0002-7095-813X

Supplementary Table S1. Search strategy (code line) for each database.

**Databases**	PubMed, WOS (Core Collection) [a], Scopus.
**Keywords**	“ballistic”, “complex”, “cycle”, “explosive”, “force”, “jump”, “plyometric”, “power”, “shortening”, “stretch”, “training”, “velocity”.
**Database fields for the search**	PubMed and WOS: all [b]; Scopus: title, abstract, keywords [b].
**Examples of search strategy code line**	PubMed: "plyometric exercise"[MeSH Terms] OR ("plyometric"[All Fields] AND "exercise"[All Fields]) OR "plyometric exercise"[All Fields] OR ("plyometric"[All Fields] AND "training"[All Fields]) OR "plyometric training"[All Fields]<br><br>WOS: ALL=plyometric and ALL=training<br><br>SCOPUS: TITLE-ABS-KEY (plyometric AND training)

[a]: except for the keyword’s “jump”, “explosive”, and “power”, searched in all WOS databases.
[b]: except for the keywords “jump”, “explosive”, and “power”, searched in the database field TITLE (a very poor efficiency was obtained in the search for results with the incorporation of other database fields).
Note: after formal database search, the list of included articles and the inclusion criteria (see Table S1) were sent to three independent world experts in the field of physical fitness, plyometric-jump training, and cerebral palsy (https://www.expertscape.com) to help identify additional relevant articles. Additionally, the experts have peer-reviewed publications in the fields of physical fitness, plyometric-jump training and/or cerebral palsy. The experts were not provided with our search strategy, to avoid biasing their own searches. Upon completion of all these steps, the databases were again consulted in search for errata or retractions of any included study.
